# Bridging the Precancerous Gap in Colorectal Cancer Through AI-Enhanced Liquid Biopsy, Endoscopy, and Digital Pathology

**DOI:** 10.3390/cancers18182940

**Published:** 2026-09-10

**Authors:** Georgios Saridakis, Dimitrios Mavroudis, John Souglakos

**Affiliations:** 1 Laboratory of Translational Oncology, Medical School, University of Crete, 70013 Heraklion, Greece; 2 Department of Medical Oncology, University General Hospital of Heraklion, 71110 Heraklion, Greece

**Keywords:** colorectal cancer, precancerous lesions, liquid biopsy, artificial intelligence, computer-aided detection, digital pathology, risk-stratified screening

## Abstract

Colorectal cancer remains a leading cause of cancer-related mortality despite a prolonged and preventable precancerous phase. This review examines how artificial intelligence and emerging biomarkers may narrow the persistent gap between detecting established cancer and identifying advanced precursor lesions. Blood-based assays integrating cell-free DNA methylation, mutations, fragmentomic patterns, and other analytes achieve encouraging sensitivity for invasive colorectal cancer but remain substantially less sensitive for advanced adenomas and sessile serrated lesions, particularly in prospective average-risk populations. Machine-learning methods can integrate complementary molecular signals, although many models remain limited by case–control designs, spectrum bias, and inadequate external validation. Computer-aided detection increases adenoma detection and reduces miss rates, but benefits for advanced neoplasia, serrated lesions, colorectal cancer incidence, and mortality remain uncertain. AI-augmented digital pathology may improve polyp classification, dysplasia grading, invasive carcinoma recognition, and case prioritization after lesion removal. The greatest clinical value of these technologies is likely to arise from coordinated use within a multimodal, risk-adapted pathway that expands screening participation, prioritizes colonoscopy for individuals at greatest risk, and preserves high-quality colonoscopy and polypectomy as the central preventive intervention.

## Simple Summary

Colorectal cancer often develops slowly from precancerous growths that can be detected and removed before cancer appears. However, current screening tests identify, mostly, established cancer more reliably than advanced adenomas and sessile serrated lesions, creating a persistent “precancerous gap.” This review examines how artificial intelligence, blood- and stool-based biomarkers, computer-assisted colonoscopy, digital pathology, and clinical risk models may help reduce this gap. The evidence suggests that these technologies are most useful when combined rather than used as replacements for colonoscopy. Noninvasive tests may increase screening participation and help identify people who need colonoscopy most urgently, while artificial intelligence may improve lesion detection and pathological classification. A coordinated, risk-adapted pathway could strengthen colorectal cancer prevention, but prospective studies are needed to confirm its clinical usefulness, cost-effectiveness, and equitable implementation.

## 1. Introduction

Colorectal cancer (CRC) remains the second leading cause of cancer-related death worldwide despite being among the most preventable malignancies because of its prolonged and well-characterized precancerous phase [1,2]. This prevention opportunity is increasingly important in the context of the rising incidence of early-onset CRC in adults younger than 50 years [2,3,4]. The conventional adenoma–carcinoma and serrated neoplasia pathways provide a window of opportunity, during which the detection and removal of precursor lesions can avert invasive disease [5,6]. Nevertheless, clinically relevant precursor lesions continue to escape screening and surveillance, and many cancers are still diagnosed after opportunities for prevention or earlier detection have been missed [3,7,8].

This discrepancy between the biological preventability of CRC and the incomplete detection of its precursors represents a persistent “precancerous gap.” Screening modalities differ not only in accessibility and cancer sensitivity but also in their capacity to prevent disease. Colonoscopy permits direct visualization and removal of precursor lesions but is limited by incomplete uptake, operator-dependent miss rates, bowel preparation, and unequal access [9]. Stool-based tests improve accessibility but remain less sensitive for advanced precancerous lesions than for invasive CRC [10]. Current blood-based assays may further expand participation, yet their sensitivity for advanced adenomas and sessile serrated lesions is substantially lower than their sensitivity for established cancer [11,12]. Greater convenience therefore does not necessarily result in equivalent cancer prevention.

Liquid biopsy and artificial intelligence (AI) may address different components of this gap. Although the most clinically mature application of circulating tumor DNA (ctDNA) in CRC lies in molecular residual disease assessment, population screening presents a fundamentally different biological and analytical task [13]. Screening assays must identify weak molecular signals from asymptomatic, frequently small, and heterogeneous lesions. AI can support high-dimensional molecular classification, real-time endoscopic lesion detection and characterization, digital pathology, and clinical risk prediction [14,15,16,17,18,19]. These applications are complementary rather than interchangeable, and none currently replaces the preventive efficacy of high-quality colonoscopy and polypectomy.

This narrative review critically evaluates AI-enhanced noninvasive biomarkers, computer-assisted colonoscopy, digital pathology, and clinical risk models across CRC screening and precursor-lesion management. Particular emphasis is placed on the distinction between early cancer detection and prevention, the methodological limitations that inflate apparent model performance, and the potential development of a multimodal and longitudinal screening pathway. Regulatory, economic, equity, and implementation challenges are also considered.

## 2. Review Methodology

This narrative review was informed by targeted searches of PubMed/MEDLINE from database inception through 21 August 2026 and relevant journal, regulatory, guideline, and professional-society sources. Search concepts included colorectal cancer, advanced adenoma, sessile serrated lesion, screening, liquid biopsy, cell-free DNA, circulating tumor DNA, methylation, fragmentomics, circulating RNA, multitarget stool testing, machine learning, computer-aided detection, computer-aided diagnosis, digital pathology, electronic health records, and risk stratification. The search was supplemented by backward reference screening of included articles and forward citation tracking of influential studies.

Titles and abstracts were screened for relevance to precursor detection and clinical management, followed by full-text assessment of potentially relevant articles. Priority was given to prospective average-risk screening cohorts, randomized trials, systematic reviews and meta-analyses, models with independent external validation, clinical guidelines, consensus statements, and regulatory documents. When multiple studies evaluated the same assay or platform, the largest and most recent independent prospective validation was prioritized. When findings were discordant, greater weight was assigned to prospective average-risk cohorts, independent external validation, randomized or pragmatic evaluations, and outcomes directly relevant to precursor detection and screening pathways. Differences in study population, reference standard, platform, and outcome definition were also considered when interpreting discordant results. Because the objective was a critical narrative synthesis rather than a systematic review, no formal meta-analysis or study-level risk-of-bias assessment was performed.

## 3. Defining the Precancerous Gap

### 3.1. Advanced Adenomas and Sessile Serrated Lesions as Prevention Targets

Colorectal carcinogenesis proceeds predominantly through conventional adenoma–carcinoma and serrated neoplasia pathways, each with precursor lesions that constitute the principal targets of cancer prevention [20,21]. Advanced adenomas are generally defined by a size of at least 10 mm, villous or tubulovillous histology, or high-grade dysplasia. Their detection is associated with a substantially increased risk of subsequent CRC compared with the absence of polyps [22,23,24,25].

The serrated pathway is characterized by *BRAF* or, less commonly, *KRAS* alterations, CpG island methylation, and widespread promoter hypermethylation and contributes to an estimated 15–30% of CRCs [21,26]. Sessile serrated lesions (SSLs) are the principal premalignant precursor in this pathway [27]. Their reported prevalence varies according to population, pathology criteria, and endoscopist performance, with estimates of approximately 5–7% in average-risk populations and higher rates among high-detecting endoscopists [27,28,29]. Large serrated polyps and SSLs with dysplasia are associated with clinically meaningful malignant potential [22,30]. Together, advanced conventional adenomas and high-risk serrated lesions represent the lesions that screening must identify and completely remove to shift CRC control from earlier detection toward prevention.

### 3.2. Limitations of Current Screening Modalities

Despite the established effectiveness of CRC screening, every available modality has limitations in the detection or accurate classification of precursor lesions. Colonoscopy misses approximately 22–26% of adenomas, with performance influenced by endoscopist expertise, bowel preparation, withdrawal technique, and lesion morphology [9,31]. SSLs are particularly vulnerable to missed detection because they are often flat, pale, mucus-capped, and located in the proximal colon [32]. Their detection varies markedly among endoscopists. The SSL detection rate has therefore emerged as a quality indicator, although its measurement and implementation remain less mature than those of the adenoma detection rate [33].

Noninvasive stool-based tests also show a sensitivity gradient across the neoplastic continuum. Fecal immunochemical testing (FIT) detects approximately 23–28% of advanced adenomas, whereas next-generation multitarget stool DNA and multitarget stool RNA tests detect approximately 43–46% of advanced precancerous lesions [10,34,35,36]. These tests perform substantially better for invasive CRC, meaning that a negative result does not reliably exclude clinically relevant precursor neoplasia.

Blood-based cfDNA screening demonstrates an even wider precancerous detection gap. In prospective average-risk screening populations, current assays detect only approximately 13% of advanced precancerous lesions despite substantially greater sensitivity for established CRC [11,37,38]. Higher estimates reported in case–control and disease-enriched studies should be interpreted cautiously because spectrum enrichment, symptomatic populations, and higher disease prevalence can inflate apparent performance [39]. This stage-dependent gradient is biologically plausible because noninvasive precursor lesions generally release far less tumor-derived material into the circulation than established cancers.

Uncertainty persists after lesion removal. Distinguishing SSLs from hyperplastic polyps can alter surveillance recommendations but remains challenging even for experienced pathologists. Interobserver agreement is moderate when classification is based on morphology alone, and approximately 11% of lesions initially classified as hyperplastic polyps are reclassified as SSLs on expert reassessment, particularly when they are larger or proximally located [40,41]. The precancerous gap therefore extends beyond lesion detection to post-resection classification and risk assignment.

### 3.3. Early Detection and Cancer Prevention

**Figure 1:**
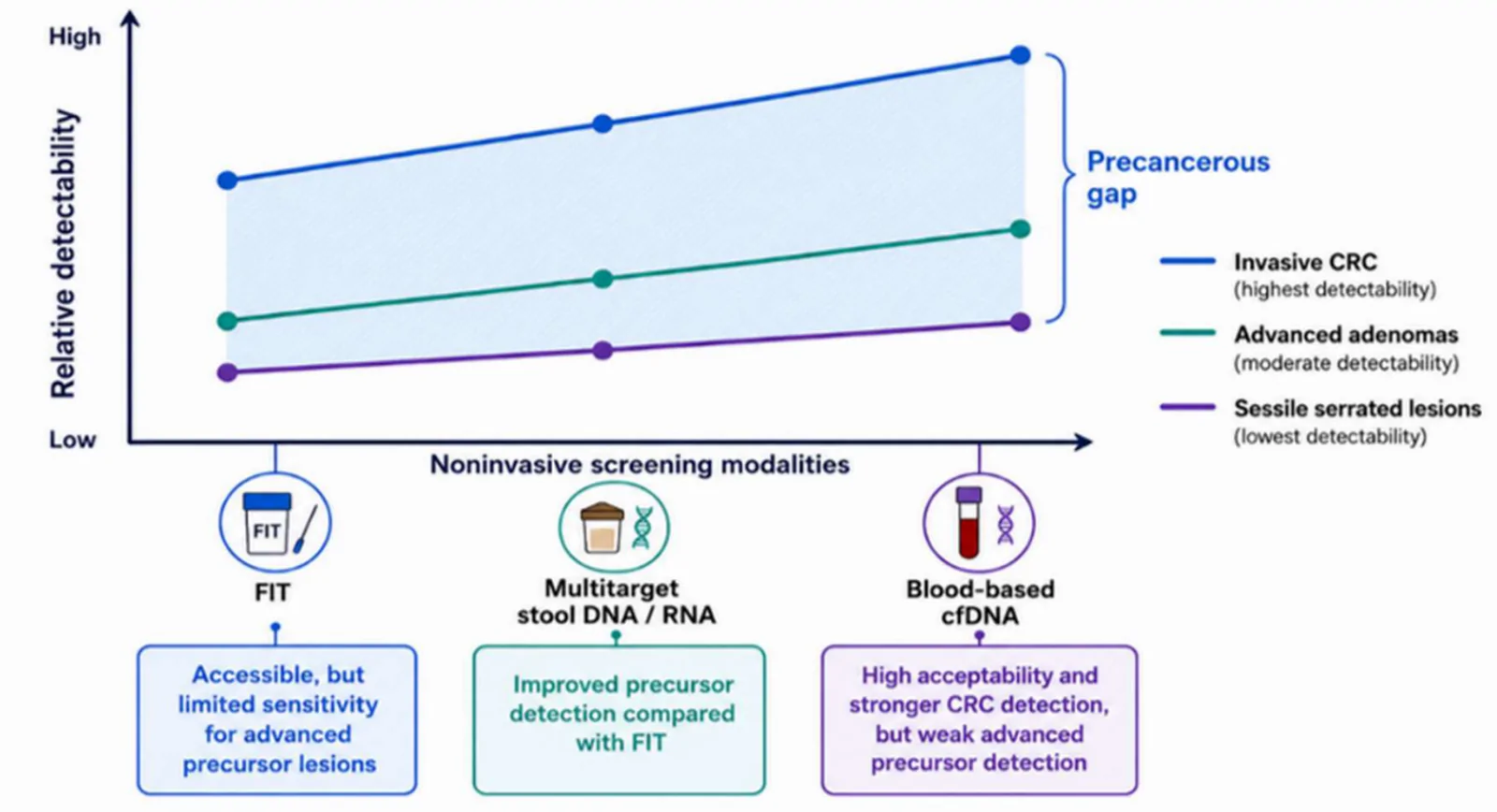
The Precancerous Gap. The figure contrasts the higher detectability of invasive CRC with the lower detectability of advanced adenomas and sessile serrated lesions across noninvasive screening modalities. Advanced adenomas occupy an intermediate position, whereas sessile serrated lesions remain the least detectable precursor category. The figure is conceptual and does not represent direct head-to-head quantitative comparisons.

Early cancer detection and cancer prevention are distinct but complementary objectives of CRC screening. Early detection identifies invasive cancer at a localized and potentially curable stage, whereas prevention requires the detection and removal of precursor lesions before malignant transformation [5,42]. Current blood-based assays predominantly address the former objective because their sensitivity increases substantially after invasive disease develops, while advanced adenomas and SSLs frequently remain undetected [11,37,38]. A strategy that improves cancer detection without predominantly identifying precursor lesions may produce a stage shift but cannot reproduce the preventive effect of lesion detection and polypectomy. Bridging the precancerous gap therefore requires a screening ecosystem that combines accessible noninvasive testing with risk stratification, high-quality colonoscopy, and accurate pathology. This differential performance is illustrated conceptually in Figure 1, while the principal strengths, precursor-lesion performance, and limitations of individual screening modalities are summarized in Table 1.

## 4. AI-Enhanced Noninvasive Biomarkers

### 4.1. Terminology and Scope of Circulating Biomarkers

Because the liquid-biopsy literature uses overlapping terminology, operational definitions are applied throughout this section. Cell-free DNA (cfDNA) refers to extracellular DNA fragments circulating in plasma, irrespective of their cellular origin, and comprises a heterogeneous mixture derived predominantly from nonmalignant cells, together with a variable tumor-derived component in individuals with cancer. Circulating tumor DNA (ctDNA) is the tumor-derived fraction of cfDNA and may contain somatic mutations, copy-number alterations, aberrant methylation, and cancer-associated fragmentation patterns. Its abundance is influenced by lesion burden, vascularity, cell turnover, and clearance and may be extremely low or undetectable in early-stage CRC and precursor lesions. In mutation-based assays, detection of a tumor-associated somatic variant supports the presence of ctDNA only after potential germline and clonal hematopoiesis-derived variants have been appropriately excluded [43,44].

Methylation and fragmentomic classifiers should be analytically distinguished from mutation-based ctDNA assays. Rather than requiring the detection of predefined somatic variants, they interrogate locus-specific or genome-wide epigenetic or structural features of cfDNA, including methylation patterns, fragment-size distributions, fragment-end motifs, nucleosome positioning, and genome-wide coverage [45]. These features may be integrated by machine-learning models to infer a cancer-associated signal. However, a positive classifier output does not necessarily constitute direct detection of a tumor-specific sequence or quantification of the ctDNA fraction [45].

In this review, “blood-based assay” is used as an umbrella term for tests performed on blood-derived specimens, including mutation-, methylation-, fragmentomic-, RNA-, and protein-based approaches. A “multi-analyte assay” refers to a test combining two or more molecular or biochemical analyte classes, such as DNA alterations, RNA transcripts, and proteins. Models that additionally incorporate clinical, demographic, or hematologic variables are more accurately described as multimodal classifiers. These distinctions are important because the biological sources of signal, analytical requirements, and potential causes of error differ across assay categories.

### 4.2. Biological Constraints of Circulating Biomarker Detection

Circulating tumor DNA (ctDNA) is released primarily through apoptosis and necrosis, with smaller contributions from active secretion and processing of cellular debris [43]. CRC has a comparatively favorable ctDNA-shedding profile among solid tumors, but detection remains strongly influenced by tumor burden, proliferative activity, vascular access, and invasive growth [44]. Whole-genome doubling and selected tumor phenotypes may further modify detectability, although these associations are not sufficiently consistent to guide screening strategies [46].

These biological determinants have direct implications for population screening. Precancerous lesions and early-stage cancers contain fewer neoplastic cells, have limited access to the systemic circulation, and generally release substantially less tumor-derived material than advanced invasive cancers [11,37,44]. Increasing analytical sensitivity cannot fully overcome the absence or extremely low concentration of a target signal in a blood sample. Machine learning can improve the recognition of subtle combinations of molecular features but cannot reliably classify a lesion when biologically informative material is absent. The primary challenge is therefore to identify signals that are released consistently before invasion occurs rather than merely to increase classifier complexity.

### 4.3. Pre-Analytical and Analytical Determinants of Assay Performance

The performance of circulating-biomarker assays is determined not only by the availability of a biological signal but also by the complete pre-analytical and analytical workflow. This is particularly important for precursor-lesion detection, in which tumor-derived material may represent only a very small fraction of total cfDNA. Variability in specimen collection, processing, storage, and extraction can alter cfDNA concentration and fragment characteristics and may therefore affect both molecular measurements and classifier output [47,48].

Plasma is generally preferred to serum because clotting can release high-molecular-weight genomic DNA from leukocytes, diluting low-frequency tumor-derived signals. Samples collected in EDTA tubes require prompt plasma separation, whereas cell-stabilizing tubes may permit longer processing intervals only under validated time and temperature conditions. Studies should standardize and report the interval from blood collection to plasma separation, transport conditions, centrifugation protocol, and procedures used to remove residual cellular material. Plasma should be aliquoted before long-term frozen storage, and repeated freeze–thaw cycles should be minimized and documented [47,48].

The extraction method influences both total cfDNA yield and recovery across fragment sizes, which is relevant because ctDNA may be relatively enriched among shorter fragments. Quality-control procedures should therefore assess extraction yield, fragment-size distribution, and contamination by high-molecular-weight genomic DNA. For mutation-based sequencing assays, performance also depends on cfDNA input, library complexity, unique molecular depth, and validated error-suppression methods, including unique molecular identifiers where appropriate. Methylation and fragmentomic assays require platform-specific quality metrics, and analytical criteria developed for variant-based assays should not be transferred uncritically to these classifiers. When only a few tumor-derived molecules are present in the sampled plasma volume, stochastic sampling may become the dominant limitation and cannot be overcome simply by increasing sequencing depth [47,49,50].

Analytical validation should be appropriate to the assay’s intended use and should empirically establish the limit of blank, limit of detection, limit of quantification where applicable, analytical specificity, accuracy, precision, stability, and reproducibility across runs, operators, reagent lots, and sites when relevant. Limits of detection should be determined for the relevant specimen type, input amount, and analytical platform rather than extrapolated across assays. Batch effects, normalization procedures, sample-acceptance criteria, failure rates, and rules for repeat testing should also be prespecified. Mutation-based assays must address potential interference from germline variants and clonal hematopoiesis through matched leukocyte analysis or appropriately validated bioinformatic filtering.

For machine-learning classifiers, the complete analytical pipeline—including specimen handling, preprocessing, feature extraction and selection, normalization, classifier version, and decision threshold—should be locked before independent validation. Re-optimization of features or positivity thresholds using validation data can inflate apparent performance and compromise reproducibility. At a minimum, studies should report specimen type and volume, collection and processing conditions, extraction method, molecular input, assay-specific quality metrics, failure rate, normalization procedure, and the prespecified classifier version and threshold [51].

### 4.4. Methylation- and Mutation-Based Approaches

DNA methylation is an early event in colorectal carcinogenesis and is therefore an attractive target for noninvasive detection [52]. A single-gene methylation assay targeting *SEPT9* demonstrated 48% sensitivity for CRC and 11% sensitivity for advanced adenomas at 91.5% specificity in a prospective screening population [53,54]. Meta-analytic estimates for CRC are higher, but they reflect heterogeneity in study design, assay generation, patient selection, and positivity thresholds [55].

Multi-gene methylation panels have shown stronger performance in developmental studies. Combined methylated *SDC2* and *SEPT9* testing achieved 80% sensitivity for stage I CRC and 47.8% sensitivity for advanced adenomas [56]. Other panels have reported higher estimates, although many were evaluated in selected case–control populations and used heterogeneous specimens and lesion definitions [57]. Machine-learning classifiers can integrate methylation across multiple genomic regions. One such model achieved an AUC of 0.96 for CRC detection, but its comparison of established CRC with controls did not establish effectiveness for precursor detection in asymptomatic screening [58].

The most clinically advanced blood-based assays combine methylation with genomic and fragmentomic features. In prospective average-risk cohorts, these platforms achieved approximately 79–83% sensitivity for CRC but only 12.5–13.2% sensitivity for advanced precancerous lesions [11,37]. These data provide the clearest evidence that clinically relevant cancer detection does not necessarily translate into prevention.

Mutation-based assays interrogate recurrent alterations in *APC*, *KRAS*, *BRAF*, *TP53*, and other genes [59]. Although highly sensitive laboratory methods can detect very low mutant allele fractions, clinical sensitivity remains constrained by low shedding and incomplete genomic coverage. A ddPCR assay targeting *RAS* and *BRAF* detected ctDNA in 45% of CRCs but only 2.6% of advanced adenomas [59]. Broader panels can identify mutations in some lower-risk adenomas, but tissue origin may be uncertain and age-related clonal hematopoiesis can generate non-colorectal variants [60]. Mutation signals are therefore more useful as components of multi-feature models than as stand-alone screening biomarkers.

### 4.5. Fragmentomics and Multi-Omic cfDNA Models

Cell-free DNA fragmentomics evaluates genome-wide fragment size distributions, end motifs, nucleosome positioning, and coverage patterns [45]. These features reflect chromatin organization, transcriptional activity, and tissue of origin and create a high-dimensional signal that is well suited to machine-learning analysis. Unlike mutation-based methods, fragmentomics does not require predefined genetic alterations.

A prospective case–control validation study reported 90.4% sensitivity for CRC and 58.3% sensitivity for advanced adenomas at 94.7% specificity [61]. Other studies of nuclear and mitochondrial fragmentomic features have reported still higher sensitivities for advanced adenomas and early-stage CRC [62,63]. These results provide proof of principle but should not be directly equated with performance in unselected population screening because many studies used disease-enriched cohorts, small precursor-lesion samples, repeated feature selection, or extensive model optimization.

Integrating fragmentation with methylation, mutations, copy-number changes, and end motifs may provide more complete molecular characterization. A multi-omic model achieved an AUC of 0.981 and 80% sensitivity for stage I CRC but was evaluated primarily in patients with established cancer and controls [64]. A separate multimodal assay detected CRC with 93% sensitivity, including 84% sensitivity for stage I disease, but detected only 23% of advanced precancerous lesions at 90% specificity [65]. The within-assay difference reinforces the distinction between early cancer detection and precursor-lesion detection.

Fragmentomic and multi-omic models therefore remain promising but developmentally immature. Validation cohorts should include advanced adenomas, SSLs, non-advanced polyps, benign gastrointestinal disorders, and negative colonoscopy findings. Performance should be reported separately according to lesion subtype, size, location, and dysplasia because aggregate CRC-versus-control discrimination is not an adequate measure of preventive capacity.

### 4.6. RNA-Based and Multi-Analyte Models

RNA-based biomarkers provide information distinct from cfDNA. Transcriptional signatures derived from peripheral blood cells, plasma, extracellular vesicles, or stool may capture both tumor-associated expression and the systemic host response. A cell-free oncRNA classifier achieved 89% sensitivity at 90% specificity for CRC, including 80% sensitivity for stage I disease [66]. A 29-gene peripheral-blood-cell signature combined with serum proteins detected CRC with 78.1% sensitivity and large adenomas with 52.3% sensitivity at 92.2% specificity [67,68]. These findings suggest that host-response signals may complement tumor-derived cfDNA, although they remain insufficient for stand-alone exclusion of advanced neoplasia.

Many exploratory blood-expression models report AUCs above 0.90, but the strongest estimates generally derive from small retrospective datasets comparing established cancers with healthy controls [69,70,71,72,73,74,75]. Differences in RNA source, extraction, sequencing, normalization, feature selection, and validation strategy limit model comparison and increase the risk of overfitting.

Stool-derived RNA models are particularly relevant because stool is in direct contact with colorectal lesions and RNA signals can be combined with occult blood. A prospective classifier incorporating eight stool RNA biomarkers, smoking status, and FIT achieved 95% sensitivity for CRC and 62% sensitivity for advanced adenomas at 85% specificity among individuals with negative colonoscopy findings [76]. A larger prospective screening study of a multitarget stool RNA test reported 94.4% sensitivity for CRC and 45.9% sensitivity for advanced adenomas, with 87.9% specificity [36]. Stool RNA–FIT combinations therefore represent one of the more promising noninvasive approaches for precursor detection, although they remain less sensitive than colonoscopy and require longitudinal outcome evaluation.

Combining analyte classes may further improve discrimination. FIT combined with methylated *SEPT9* achieved greater CRC discrimination than either component alone [77]. Other composite models have incorporated methylation, serum tumor markers, hematologic variables, and stool blood testing [78,79]. However, small increases in AUC for established CRC should not be mistaken for clinically meaningful progress. Multi-analyte development should prioritize advanced adenoma and SSL detection at an acceptable false-positive colonoscopy rate.

### 4.7. Analytical Validity, Clinical Validity, and Clinical Utility

Evaluation of a noninvasive CRC screening biomarker should distinguish analytical validity, clinical validity, and clinical utility because these represent sequential but non-interchangeable levels of evidence [80,81].

Analytical validity concerns whether the assay measures its intended molecular signal accurately, reproducibly, and robustly under the conditions of its intended use. It encompasses analytical sensitivity and specificity, accuracy, precision, reproducibility, interference, stability, and performance under the pre-analytical conditions described in Section 4.3. Analytical validity is necessary but does not establish that the measured signal identifies clinically relevant colorectal neoplasia [47,81].

Clinical validity concerns whether the test identifies the target condition in its intended-use population. For CRC screening, clinical validity is most convincingly established in asymptomatic, average-risk individuals undergoing colonoscopy, with histopathologic confirmation of detected lesions as the reference standard. Relevant measures include sensitivity, specificity, positive and negative predictive values, and calibration when a probabilistic risk score is reported. Performance should be reported separately for CRC, advanced adenomas, sessile serrated lesions, nonadvanced polyps, and negative colonoscopy findings and, where possible, according to lesion size, location, and degree of dysplasia. Case–control discrimination between established cancers and healthy controls is insufficient to establish clinical validity for population screening [80,81].

Clinical utility concerns whether incorporating the test into a screening pathway improves patient-relevant outcomes compared with existing strategies without disproportionate harm or cost. Relevant implementation outcomes include screening uptake, adherence, and completion of diagnostic colonoscopy after a positive result. Patient-relevant outcomes include the detection and removal of advanced precursor lesions, interval cancer incidence, stage distribution, CRC incidence, and mortality, balanced against false-positive colonoscopies, false reassurance following false-negative results, overdiagnosis, and cost. Diagnostic accuracy or stage shift alone does not establish preventive benefit, and prospective evaluation of the complete screening pathway remains necessary [80].

**Figure 2:**
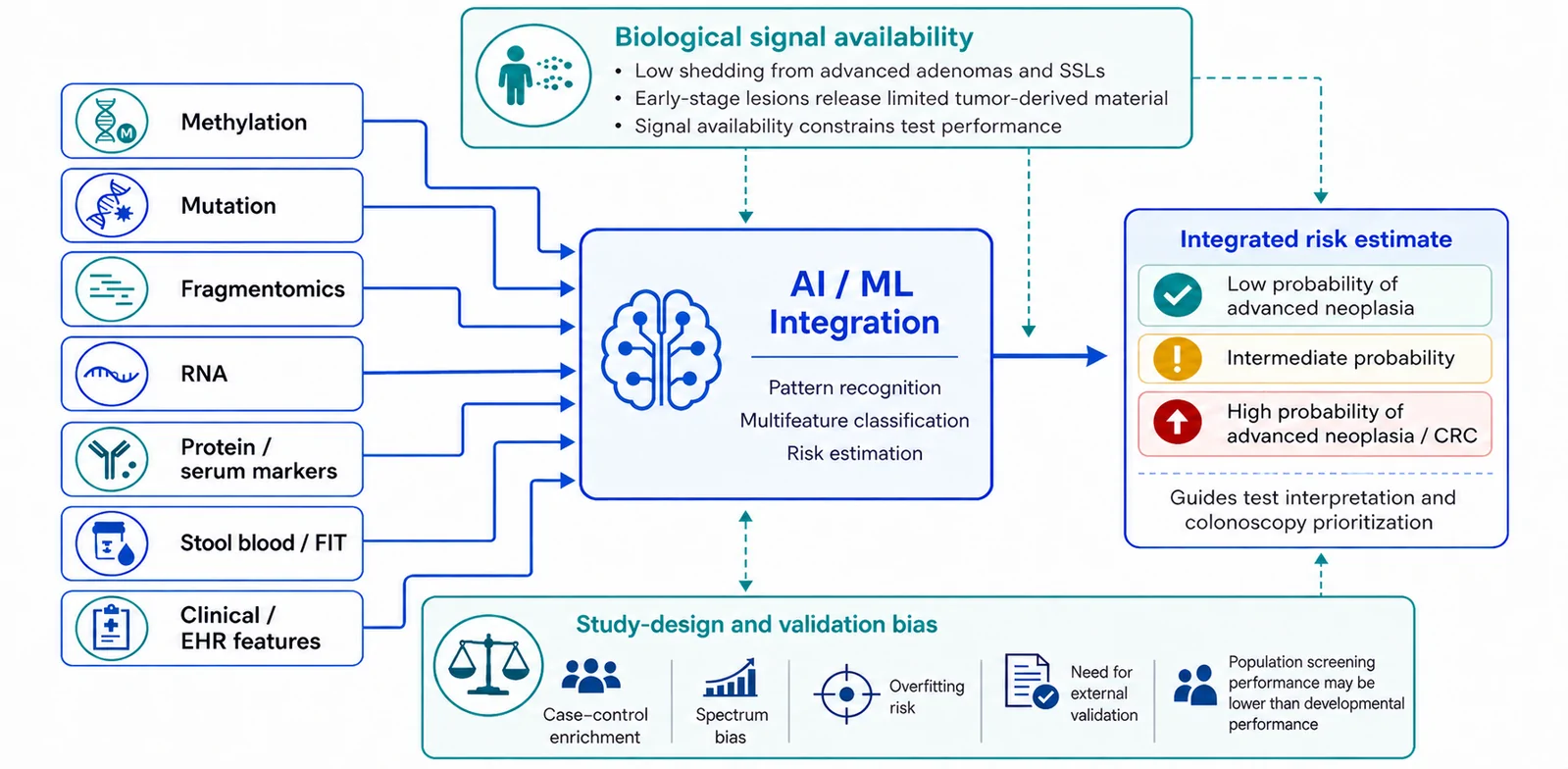
AI-enhanced noninvasive biomarker interpretation. Complementary methylation, mutation, fragmentomics, RNA, protein/serum markers, stool blood/FIT, and clinical/EHR features are integrated by an AI/ML module to generate a composite risk estimate for advanced neoplasia/colorectal cancer and guide test interpretation and colonoscopy prioritization. Model performance remains constrained by biological signal availability, particularly the limited shedding of advanced precursor lesions, and by methodological factors such as case–control enrichment, spectrum bias, overfitting, inadequate external validation, and potentially lower performance in population-screening settings. Solid arrows indicate the direct flow of information from the input feature domains to the AI/ML integration module and subsequently to the integrated risk estimate, whereas dashed arrows indicate contextual or methodological influences—not direct data flow—related to biological signal availability and study-design/validation factors.

Across mutation-, methylation-, fragmentomic-, transcriptomic-, and multi-analyte approaches, performance remains substantially stronger for established CRC than for advanced precursor lesions. In prospective population-based screening studies, pooled sensitivity of ctDNA-based blood tests was approximately 72% for CRC but only 13% for advanced precancerous lesions [38]. Thus, although individual assays may demonstrate acceptable analytical performance and clinical validity for CRC detection, their clinical validity for precursor detection remains limited, and direct preventive utility—particularly reductions in CRC incidence and mortality—has not been established. The 2026 American Cancer Society guideline accordingly positions blood-based testing as a non-preferred option for average-risk individuals who decline or do not complete preferred stool-based testing or visual examination, rather than as a replacement for lesion-directed prevention [82]. A positive blood-based test requires timely diagnostic colonoscopy, while the potential use of these assays as an adjunct to risk stratification remains investigational. The interaction between biological detectability, multimodal signal integration, and validation bias is illustrated in Figure 2, while representative liquid-biopsy and multi-analyte strategies are compared in Table 2.

## 5. AI-Assisted Endoscopy

### 5.1. Computer-Aided Detection

Computer-aided detection (CADe) systems analyze colonoscopy video in real time and mark suspected lesions to attract the endoscopist’s attention [83]. A meta-analysis involving more than 30,000 participants found that CADe increased the polyp detection rate from 47.9% to 56.1% and the adenoma detection rate (ADR) from 36.7% to 44.7%, while reducing the adenoma miss rate from 35.3% to 16.1% [15]. Advanced colorectal neoplasia detection increased more modestly, from 11.5% to 12.7% [15].

Platform-specific analyses suggest heterogeneity rather than a uniform CADe class effect. One network meta-analysis of 48 randomized trials involving 38,986 participants found higher SSL detection rates with ENDO-AID (RR 1.36, 95% CrI 1.03–1.79) and GI Genius (RR 1.25, 95% CrI 1.08–1.46) [84]. Conversely, another network meta-analysis of 48 randomized trials and 34,106 participants found no statistically significant improvement in advanced adenoma or SSL detection for any evaluated platform [85]. These discordant findings, together with limited direct head-to-head evidence and potential differences between software versions, preclude firm conclusions regarding the superiority of individual CADe systems. Current clinical guidance therefore does not make a definitive recommendation for or against routine CADe use because certainty remains very low for clinically important outcomes [14]. The central unresolved question is whether improvements in surrogate detection metrics translate into fewer interval cancers, lower CRC incidence, or reduced mortality.

### 5.2. Computer-Aided Diagnosis

Computer-aided diagnosis (CADx) systems characterize detected polyps in real time and may support optical diagnosis and resect-and-discard strategies for diminutive lesions [83]. Earlier meta-analyses reported high standalone accuracy, particularly when CADx was combined with narrow-band imaging [86]. More recent comparative analyses, however, have not demonstrated a consistent incremental advantage over optical diagnosis by experienced endoscopists [87,88,89].

CADx performance also varies by anatomical location and lesion type. Accuracy is lower in the proximal colon, where SSLs predominate, and binary neoplastic versus non-neoplastic classification does not capture all subtypes relevant to resection and surveillance [90,91,92]. CADx should therefore remain a decision-support tool rather than an autonomous determinant of management until prospective implementation studies demonstrate consistent superior performance in routine practice.

### 5.3. Serrated and Non-Polypoid Lesions

SSL detection remains a critical gap in AI-assisted colonoscopy. A meta-analysis of 28 randomized trials found no significant overall improvement in SSL detection or SSL miss rate with CADe [93]. Other pooled analyses and individual pragmatic trials have reported more favorable results, including improved SSL detection in selected systems and screening settings [17,84,94,95,96]. This heterogeneity likely reflects differences in patient populations, lesion prevalence, mucosal exposure, device architecture, and study methodology.

The intrinsic morphology of SSLs also limits performance. These lesions are often flat, pale, mucus-capped, and located in the proximal colon, reducing visual conspicuity for both endoscopists and algorithms [32,94]. In contrast, evidence for non-polypoid lesions is somewhat more consistent, with increased detection of flat adenomas in meta-analyses and pragmatic randomized data [94,95]. CADe therefore improves recognition of subtle lesions overall, but its specific ability to close the SSL detection gap remains unproven.

### 5.4. Workflow, Over-Detection, and Clinical Effectiveness

CADe adds only a small amount to withdrawal time, with pooled mean differences of approximately 0.15–0.57 min [14,15,93]. However, it increases the resection of non-neoplastic polyps, with relative increases of approximately 11–39% [15,93]. Additional polypectomy, pathology, procedural risk, and surveillance may therefore offset part of the benefit obtained through greater detection.

Routine-practice studies have sometimes reported smaller benefits than conventional randomized trials, although a large pragmatic cluster-randomized study found that an ADR benefit could be retained at scale [97,98,99]. CADe appears to augment performance across experience levels rather than merely compensating for inexperienced operators [93,99]. Potential deskilling remains a concern, but current studies are inconsistent, with one observational study suggesting lower subsequent unassisted ADR and a prospective trial finding neither upskilling nor deskilling [100,101].

ADR is strongly associated with lower post-colonoscopy CRC incidence and mortality, but the preventive value of CADe cannot be inferred directly from its effect on ADR [33]. Much of the additional detection involves diminutive adenomas, while benefits for advanced adenomas and SSLs remain smaller or inconsistent [14,15,93]. No randomized trial has yet demonstrated a reduction in CRC incidence or mortality. The appropriate evaluation of CADe must therefore balance missed clinically relevant lesions against unnecessary intervention and surveillance burden. The principal evidence and limitations of AI-assisted colonoscopy are summarized in Table 3.

**Table 3:** Evidence and limitations of AI-assisted colonoscopy.

Application	Best-Supported Effect	Less Certain Effect	Potential Harm or Limitation	References
CADe	Increases ADR and polyp detection; reduces adenoma miss rate	Advanced adenoma, SSL, interval CRC, incidence, and mortality	Additional diminutive and non-neoplastic polypectomy	[14,15,84,85,93]
CADx	High standalone optical classification accuracy under selected conditions	Incremental benefit over experienced endoscopists	Location- and lesion-dependent performance; limited subtype resolution	[83,86,87,88,89,90,91,92]
Serrated-lesion detection	Possible benefit in selected platforms and settings	Consistent class-wide improvement in SSL detection	Flat morphology, mucus cap, and proximal location	[17,84,85,93,94,95,96]
Routine implementation	Small withdrawal-time increase and scalable ADR benefit in some pragmatic studies	Long-term prevention and cost-effectiveness	Alert fatigue, over-detection, and possible deskilling	[97,98,99,100,101]

Abbreviations: ADR, adenoma detection rate; CADe, computer-aided detection; CADx, computer-aided diagnosis; SSL, sessile serrated lesion.

## 6. AI-Augmented Digital Pathology

Digital pathology captures spatial and morphological information directly from tissue and therefore complements the systemic signals obtained through noninvasive biomarkers. Deep-learning analysis of routine hematoxylin and eosin slides can support polyp classification, dysplasia grading, invasive carcinoma recognition, and case prioritization. Within CRC prevention, its most immediate role is to standardize the post-detection characterization of precursor lesions, particularly SSLs and morphologically overlapping polyp subtypes. These applications can be grouped according to their intended clinical role as autonomous multiclass classification, AI-assisted decision support, or binary triage and case prioritization. At present, decision support and triage appear more immediately applicable than fully autonomous diagnosis because they preserve pathologist oversight while targeting diagnostic consistency and workflow efficiency.

### 6.1. Polyp and Sessile Serrated Lesion Classification

A whole-slide deep-learning model externally evaluated on 238 slides from 24 institutions classified tubular adenomas, tubulovillous or villous adenomas, hyperplastic polyps, and SSLs with performance comparable to local pathologists [16]. Errors were concentrated in morphologically overlapping categories, particularly adenomatous subtypes and the distinction between hyperplastic polyps and SSLs.

Human–AI interaction may be more clinically relevant than standalone accuracy. Accordingly, the most clinically relevant comparator is often pathologist performance with AI versus without AI, rather than standalone AI versus a pathologist. In a crossover study involving 15 pathologists and 100 slides, an interactive system increased classification accuracy from 73.9% to 80.8%, with the greatest improvement for tubulovillous and villous adenomas [102]. Dedicated SSL systems have achieved performance comparable with expert pathologists and superior to less experienced readers, while interpretable approaches based on glandular orientation have demonstrated the value of combining deep features with explicit architectural measurements [103,104]. These findings support the near-term use of AI as a second reader or decision-support tool rather than as an autonomous diagnostic system, with the pathologist retaining final responsibility.

### 6.2. Dysplasia, Invasive Carcinoma, and Case Prioritization

AI models can distinguish high-grade dysplasia and invasive carcinoma from lower-risk lesions, a clinically important task when choosing between endoscopic and surgical management. A four-class whole-slide model achieved 95.5% internal accuracy and 98.5% accuracy for adenocarcinoma detection in an external cancer cohort, although the external dataset did not evaluate the complete multiclass framework [105]. Multicenter tissue-segmentation systems have also demonstrated high slide-level tumor sensitivity across different pathology departments [106].

Accordingly, case prioritization may provide a more immediate workflow benefit than autonomous classification. A system trained on more than 15,000 whole-slide images and externally validated in more than 10,000 cases assigned 98.9% of polyps with high-grade dysplasia or adenocarcinoma to specialist review while identifying 83.0% of normal or low-grade dysplastic polyps as not requiring priority review [107]. Other systems have used weakly supervised learning or binary high-risk versus low-risk classification to prioritize colorectal biopsies according to pathological severity [108,109]. For this use case, sensitivity for high-risk slides, false-negative rates, and turnaround time are more clinically informative than overall classification accuracy [107,108,110]. However, a decline from 93.4% internal accuracy to 84.9% external accuracy in another system illustrates the continuing problem of domain shift [111].

### 6.3. Clinical Scope and Implementation Limitations

Most digital pathology models have been developed retrospectively in selected or diagnostically enriched datasets. Differences in fixation, sectioning, staining, scanners, image resolution, reference standards, and local diagnostic criteria may reduce performance after deployment. Direct comparisons of reported accuracy are therefore unreliable when studies differ in class prevalence, unit of analysis, and handling of uncertain cases.

Prospective multicenter studies should evaluate not only standalone model accuracy but also pathologist performance with and without AI assistance, turnaround time, clinically important error rates, surveillance recommendations, workload and patient outcomes. A human-in-the-loop workflow may improve consistency, support second reading, and prioritize potentially high-risk or discordant slides while preserving the pathologist’s final diagnostic responsibility [102]. However, it may also introduce automation bias, over-reliance on algorithmic output, alert fatigue, and possible deskilling. Risk mitigation should include transparent presentation of model uncertainty, clinician override, predefined decision thresholds, monitoring of false-negative and discordant cases, and periodic reassessment of both model and unaided human performance [110]. Human–AI interaction is especially relevant for SSLs because morphological overlap and moderate interobserver agreement remain important sources of variability [40].

**Figure 3:**
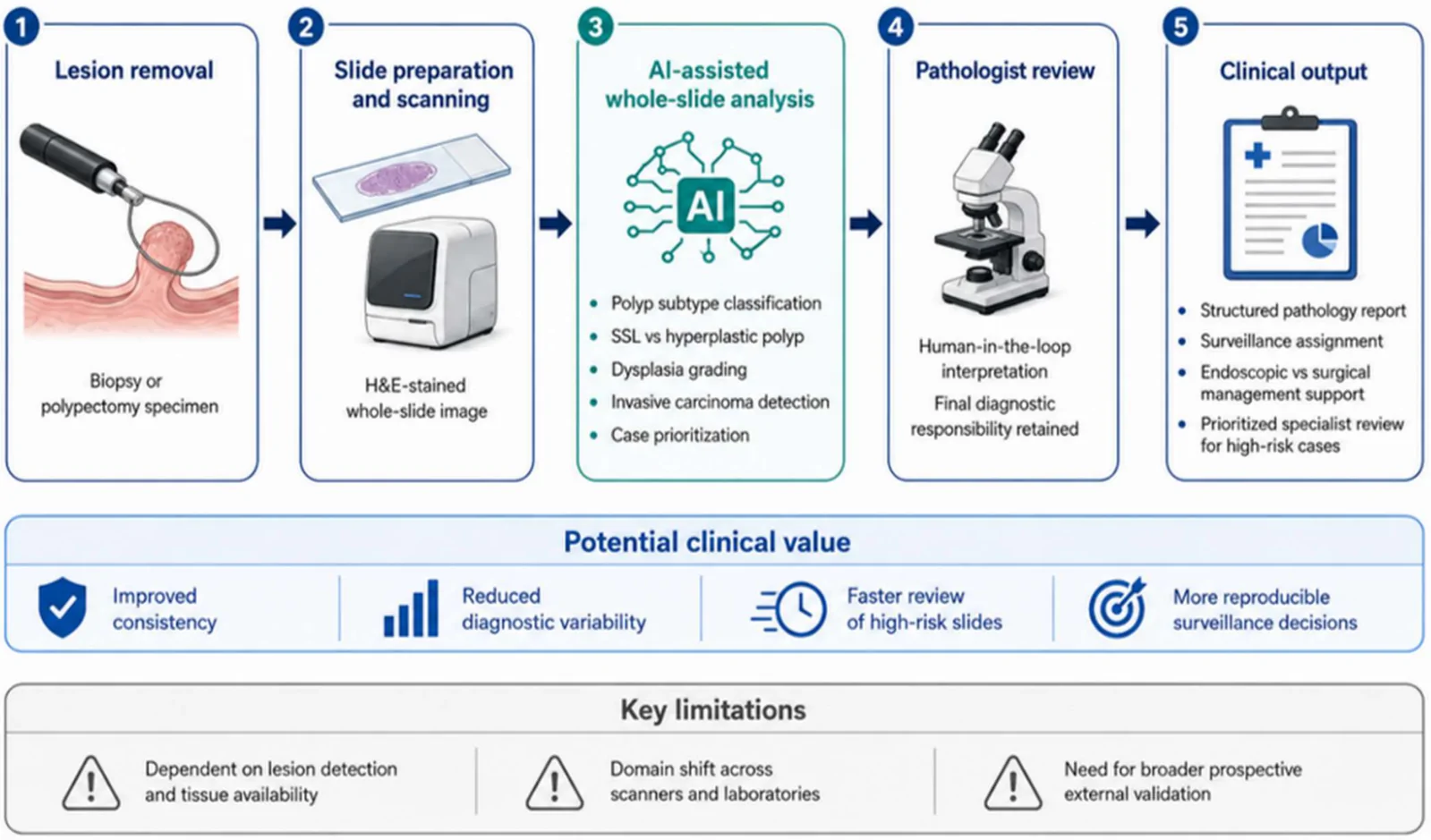
AI-augmented digital pathology after lesion removal. Following biopsy or polypectomy, whole-slide image analysis may support polyp classification, differentiation of sessile serrated lesions from hyperplastic polyps, dysplasia grading, invasive carcinoma detection, and case prioritization. AI remains embedded within a human-in-the-loop workflow in which the pathologist retains final diagnostic responsibility.

Digital pathology also depends on tissue availability. A lesion must first be detected, biopsied or resected, and processed. Pathology AI therefore cannot replace population screening. Its contribution lies downstream through better classification, dysplasia grading, case prioritization, and more reproducible assignment of surveillance and treatment pathways. The downstream workflow of AI-augmented digital pathology is illustrated in Figure 3, while representative clinical applications and their principal limitations are summarized in Table 4.

## 7. Risk Stratification and an Integrated Screening Pathway

### 7.1. Electronic Health Record and Clinical Risk Models

Electronic health record (EHR) data offer a scalable source of predictive information without additional specimen collection. Models using routine laboratory values, diagnoses, medications, demographic characteristics, and longitudinal trajectories may identify individuals at increased risk and support targeted deployment of molecular tests or colonoscopy.

A multimodal EHR model incorporating complete blood count, metabolic laboratory results, diagnostic codes, and medication records was trained on 87,825 screening colonoscopies and achieved AUROCs of 0.93 for CRC and 0.98 for CRC or advanced adenoma in an independent cohort of 21,957 colonoscopies [112]. A nationwide model developed from more than 1.1 million screening-eligible individuals enriched CRC incidence more than eightfold in the highest 1% of predicted risk and further stratified risk among individuals with a positive stool blood test [113].

Simpler models based mainly on complete blood count and demographic variables show more modest discrimination but may be easier to integrate continuously into routine care. At 97% specificity, one algorithm classified 3% of a community population as requiring investigation and identified 35% of CRCs diagnosed during the following six months [114]. A separate interpretable model based on blood-count-derived variables and age achieved an AUC of approximately 0.77 [115].

Clinical risk scores also remain relevant. A 13-variable model stratified asymptomatic individuals into advanced-neoplasia risk groups of 1.5%, 7.06%, and 27.26% [116]. A neural network using self-reported health information reduced the proportion of CRC cases classified as low risk compared with age-based recommendations [117]. A multicenter pre-colonoscopy model achieved an AUROC of 0.84 and substantially reduced the number needed to scope among individuals with a predicted CRC probability of at least 5% [118]. Meta-analytic evidence supports the discriminatory potential of machine-learning models for CRC and advanced polyps but also identifies considerable heterogeneity and risk of bias [119].

The clinical value of risk models depends on calibration, transportability, workflow integration, and their effect on screening completion and advanced-neoplasia detection. High AUCs obtained from CRC-versus-control comparisons should not be assumed to translate into effective population triage. Models must be validated across diverse populations and should be evaluated for differential performance by age, sex, race, ethnicity, socioeconomic status, and healthcare setting [120].

### 7.2. Current Screening Context

The 2026 American Cancer Society guideline reaffirms CRC screening from age 45 through age 75 for average-risk adults with a life expectancy greater than 10 years, with individualized decisions between 76 and 85 years. Visual examinations and stool-based tests remain the preferred screening options. Next-generation multitarget stool DNA and multitarget stool RNA tests are recommended at three-year intervals, whereas blood-based cfDNA testing is positioned as a non-preferred option for individuals who decline or do not complete preferred screening. Any positive non-colonoscopy screening result should be followed by timely diagnostic colonoscopy, preferably within six months [82].

### 7.3. Proposed Dynamic, Risk-Adaptive, AI-Integrated CRC Screening Pathway

**Figure 4:**
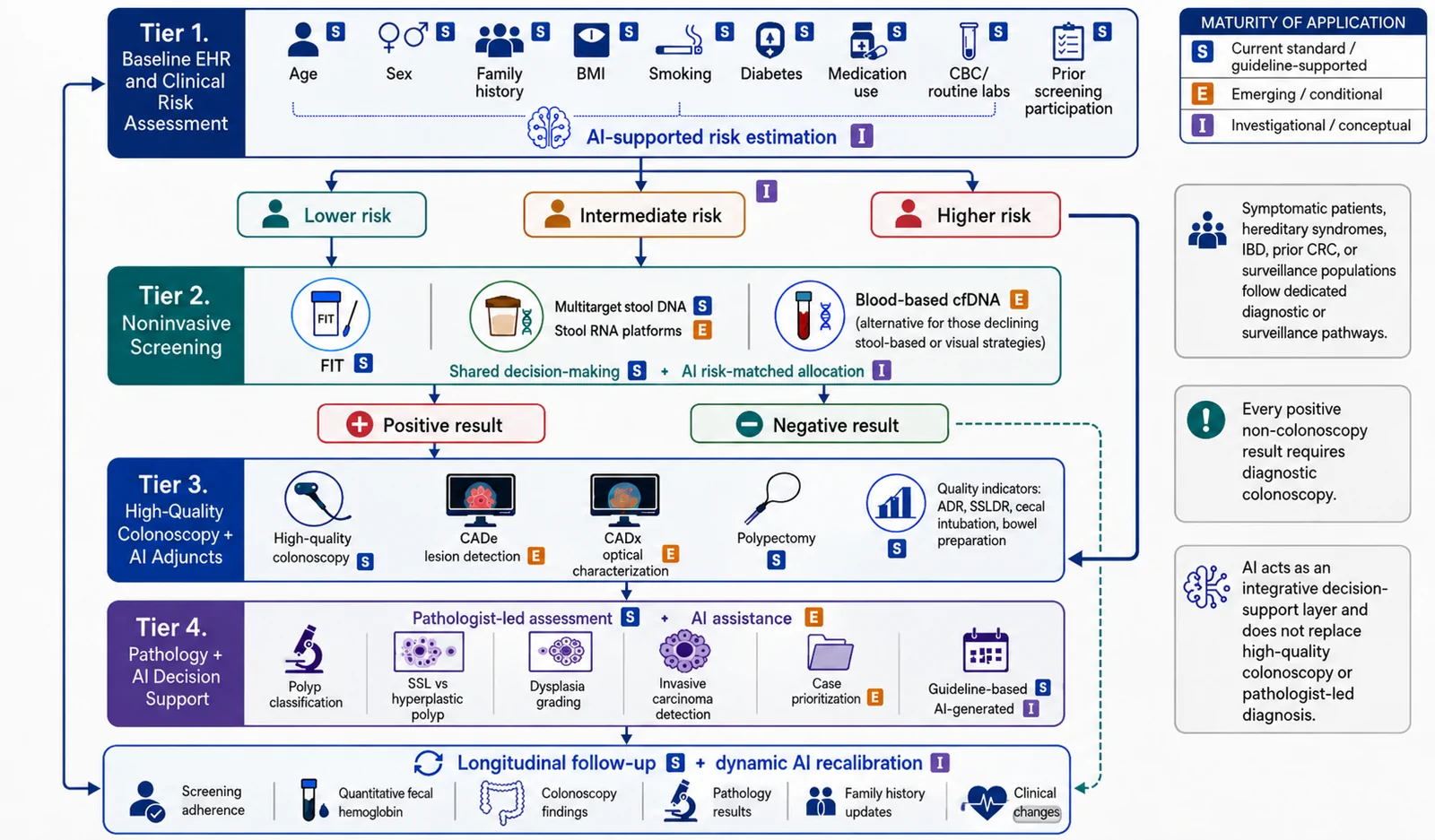
Proposed AI-enhanced, risk-adapted colorectal cancer screening pathway. Components are designated as current standard or guideline-supported [S], emerging or conditional [E], or investigational or conceptual [I]. The conceptual framework comprises baseline clinical and EHR-based risk assessment, risk-matched noninvasive screening, quality-assured AI-assisted colonoscopy, and AI-augmented digital pathology. Solid arrows indicate the primary forward flow through the risk-stratified screening pathway, whereas dashed arrows indicate longitudinal feedback and dynamic risk updating. Positive non-colonoscopy results and higher baseline risk lead to colonoscopy, while screening adherence, quantitative fecal hemoglobin, colonoscopy findings, pathology results, and changes in clinical or family history support longitudinal risk updating. The pathway is conceptual and requires prospective validation.

Drawing on the evidence summarized in this review, we propose a dynamic, risk-adaptive, AI-integrated screening pathway designed to combine the accessibility of noninvasive testing with the preventive capacity of high-quality colonoscopy. Rather than functioning as an independent screening modality, AI is positioned as an integrative layer that supports eligibility triage, individualized risk estimation, interpretation of noninvasive biomarkers, endoscopic lesion detection, histopathologic classification, and longitudinal reassessment. The proposed framework comprises four interconnected tiers together with cross-cutting mechanisms for dynamic risk recalibration, quality assurance, equity, and program-level monitoring. It should be regarded as a conceptual model requiring prospective validation rather than as an established clinical algorithm. An overview of the proposed pathway is presented in Figure 4. To distinguish established clinical practice from proposed future applications, pathway components are classified as current standard or guideline-supported, emerging or conditional, and investigational or conceptual. These categories refer to the maturity of each application within the screening pathway rather than to the intrinsic performance of the underlying technology.

#### 7.3.1. Eligibility Triage and Baseline Risk Stratification

The pathway begins by distinguishing average-risk screening from diagnostic evaluation and post-treatment or post-polypectomy surveillance. Asymptomatic adults meeting age- and health-based screening criteria would enter the average-risk pathway, whereas individuals with gastrointestinal symptoms, inflammatory bowel disease, hereditary CRC syndromes, previous CRC, or a history of polyps requiring surveillance would be directed to dedicated diagnostic or surveillance pathways. Screening would generally begin at 45 years of age, while continuation after 75 years would be individualized according to previous screening, comorbidity, life expectancy, and patient preference [120,121]. Established stool-based and direct-visualization strategies should remain the preferred entry options, while blood-based testing may be considered for individuals who decline or do not complete these approaches [12].

Among individuals entering the average-risk pathway, baseline risk would be estimated using routinely available demographic and clinical data. Potential predictors include age, sex, family history, body mass index, smoking, diabetes, medication use, previous screening participation, complete blood count parameters, and longitudinal electronic health record data. Risk models should generate graded rather than binary outputs, allowing individuals to be classified into clinically interpretable low-, intermediate-, or high-risk categories. These categories could influence the initial screening modality and the intensity of subsequent reassessment, although decision thresholds and their effects on clinical outcomes require prospective validation [112,113,114,115,116,117,118,119,120].

Polygenic risk scores may provide an additional enrichment layer when they are validated and equitably available. Models combining genetic, environmental, and clinical variables can improve risk differentiation and generate substantially different estimated ages for screening initiation across risk strata [122,123]. However, these approaches have not established prospectively validated thresholds for routine clinical decision-making, and many polygenic models have been developed predominantly in populations of European ancestry. Polygenic risk scores should therefore remain optional components rather than gatekeepers for screening eligibility or colonoscopy access. Their use should be conditional on external validation, calibration across diverse populations, cost-effectiveness, and evidence that they improve patient-relevant outcomes. Current expert guidance continues to prioritize age, hereditary predisposition, family history, and previous colonoscopy findings as the principal determinants of CRC risk stratification [120].

#### 7.3.2. Risk-Matched Noninvasive Screening

Individuals who do not proceed directly to colonoscopy would be offered noninvasive screening according to baseline risk, preventive capacity, accessibility, and patient preference. Rather than treating all noninvasive modalities as interchangeable, the proposed pathway incorporates modality-risk matching. Individuals at higher predicted risk of advanced neoplasia could preferentially be offered tests with greater sensitivity for advanced precancerous lesions or proceed directly to colonoscopy, whereas individuals at lower risk could continue screening with less invasive modalities. These assignments remain investigational and should not restrict access to established screening options.

Stool-based tests currently provide a stronger preventive rationale than blood-based assays because they demonstrate greater sensitivity for advanced precancerous lesions. FIT detects approximately 23–28% of advanced adenomas, while multitarget stool DNA and multitarget stool RNA assays detect approximately 43–46% of advanced precancerous lesions [10,34,35,36]. Although these sensitivities remain considerably lower than that of colonoscopy, stool-based testing can identify both invasive cancer and a clinically relevant proportion of precursor lesions and should therefore remain the preferred noninvasive approach for most individuals.

Current blood-based cfDNA assays detect approximately 79–83% of established CRCs but only 12.5–13.2% of advanced precancerous lesions in prospective average-risk cohorts [11,37,38]. Blood-based screening should consequently be positioned primarily as an alternative for individuals who decline or do not complete stool-based screening or direct visualization. Its principal current value lies in expanding participation and detecting established cancer rather than reliably preventing CRC through precursor-lesion identification [12].

Quantitative fecal hemoglobin concentration may provide an additional method for personalizing FIT-based screening. Rather than treating all negative FIT results as equivalent, the measured fecal hemoglobin concentration could be retained as a continuous marker of future neoplasia risk. In a population-based cohort of more than 3.5 million participants, increasing fecal hemoglobin concentrations were associated with progressively greater risks of colorectal neoplasia and CRC mortality. A model-based personalized interval strategy was estimated to reduce FIT use by 49% and colonoscopy use by 28% compared with universal biennial screening while preserving modeled effectiveness [124]. These findings support the potential use of shorter intervals for individuals with higher subthreshold concentrations and longer intervals for those with undetectable or very low concentrations. However, this approach is not yet an established standard and requires prospective validation, including evaluation in ongoing personalized FIT screening trials [125].

Every positive non-colonoscopy screening result would trigger referral for diagnostic colonoscopy. Completion of this step should be considered part of the screening process rather than a separate episode of care. The proportion of individuals completing colonoscopy and the interval between a positive test and colonoscopy should be monitored as pathway-level quality indicators, with automated navigation and alerts used to minimize clinically important delays.

Negative results would lead to repeat screening at intervals determined by the selected modality and modified, where prospectively validated, by baseline risk, previous results, fecal hemoglobin trajectory, and changes in clinical status. Screening participation should itself be incorporated into risk management because an acceptable completed test may provide greater population benefit than a theoretically superior modality that is repeatedly declined.

#### 7.3.3. Quality-Assured AI-Assisted Colonoscopy

Individuals classified as high risk at baseline and those with positive noninvasive tests would undergo high-quality colonoscopy. Computer-aided detection may be incorporated as an adjunct to careful mucosal inspection, particularly in settings where baseline detection performance is suboptimal. Meta-analyses of randomized trials demonstrate that CADe increases adenoma detection and reduces adenoma miss rates [14,15,17,84,85,93]. Nevertheless, current guidance does not provide a definitive recommendation for or against routine CADe use because evidence remains uncertain for advanced neoplasia, sessile serrated lesions, interval CRC, mortality, and cost-effectiveness [14].

The benefit of CADe should not be equated automatically with greater cancer prevention. Much of the incremental detection observed in trials has involved diminutive adenomas, whereas improvements in advanced adenoma and sessile serrated lesion detection have been smaller or inconsistent [14,15,84,85,93]. Additional detection may also increase non-neoplastic polypectomy and surveillance intensity without established long-term benefit. CADe should therefore be evaluated according to clinically relevant lesion yield, baseline endoscopist performance, downstream management, and patient-important outcomes rather than ADR improvement alone.

Accordingly, CADe should be framed as a supportive technology rather than a replacement for endoscopist competence, adequate bowel preparation, careful withdrawal, and systematic inspection of the proximal colon. Its effectiveness should be evaluated in relation to lesion subtype, additional polypectomy burden, surveillance consequences, and long-term clinical outcomes.

Computer-aided diagnosis may further support real-time optical characterization of diminutive polyps. However, its clinical application should be conditional on prospectively validated performance thresholds and local quality assurance. Current systems may have limited specificity, particularly in the proximal colon, and binary neoplastic versus non-neoplastic outputs may not provide sufficient granularity to distinguish conventional adenomas, hyperplastic polyps, and sessile serrated lesions reliably [83,86,87,89,91,92]. CADx-assisted resect-and-discard or diagnose-and-leave strategies should therefore be implemented only when established negative predictive value, surveillance agreement, and competency requirements are consistently met.

The pathway should distinguish examination-level quality from endoscopist- and program-level quality. A specific colonoscopy should be considered technically complete only when the cecum has been reached, bowel preparation is adequate, mucosal visualization is satisfactory, and identified lesions are appropriately characterized and removed. Inadequate preparation or incomplete examination should lead to earlier repeat colonoscopy according to the nature of the deficiency.

By contrast, ADR, sessile serrated lesion detection rate, cecal intubation rate, and preparation adequacy are longitudinal performance measures rather than characteristics of a single negative examination. Current quality targets include an overall ADR above 35%, sessile serrated lesion detection rate above 6%, cecal intubation in more than 95% of examinations, and adequate bowel preparation in more than 90% of procedures [33]. Persistently suboptimal performance should trigger remediation, additional training, workflow review, or referral rather than automatic repetition of every colonoscopy performed by that endoscopist.

#### 7.3.4. AI-Augmented Pathology and Surveillance Assignment

All resected lesions would undergo standard histopathologic evaluation, supplemented where available by AI-based image analysis. The principal role of AI at this stage is not population screening but downstream diagnostic standardization. Potential applications include differentiation of conventional adenomas, hyperplastic polyps, and sessile serrated lesions, grading of dysplasia, identification of invasive carcinoma, detection of diagnostically discordant cases, and prioritization of slides requiring specialist review.

Large-scale pathology classifiers have demonstrated the potential to reduce workload while preserving high sensitivity for clinically important lesions. One externally validated system assigned 98.9% of polyps containing high-grade dysplasia or adenocarcinoma to specialist review while identifying 83% of normal or low-grade dysplastic polyps as not requiring priority review [107]. In a crossover study involving 15 pathologists and 100 colorectal polyp slides, AI-assisted assessment increased classification accuracy from 73.9% to 80.8% compared with conventional microscopic evaluation [102]. These findings support a human-in-the-loop model in which AI assists triage and interpretation while the pathologist retains final diagnostic responsibility.

The final pathology report should be integrated with colonoscopy findings to assign a guideline-concordant surveillance pathway. Recommended intervals should account for lesion number, size, histologic subtype, dysplasia, anatomical location, completeness of resection, bowel preparation, and overall examination quality rather than relying on a simplified low- versus high-risk classification [6,120].

Structured AI-supported pathology reports could automatically transfer relevant lesion characteristics into the electronic health record and generate a proposed surveillance interval for clinician verification. Such systems may reduce both premature surveillance in low-risk individuals and delayed follow-up in those with high-risk lesions. Nevertheless, algorithm-generated recommendations should remain transparent, traceable to the applicable guideline, and subject to clinical review, particularly when pathology, endoscopic appearance, and resection characteristics are discordant.

#### 7.3.5. Dynamic Reassessment, Equity, and Program-Level Monitoring

The pathway should operate as a longitudinal surveillance system rather than as a one-time sequence of screening tests. After each screening cycle, risk estimates would be updated using newly available information, including screening adherence, quantitative fecal hemoglobin values, colonoscopy findings, pathology results, examination quality, interval laboratory changes, and updated family history. Individuals without detected lesions would remain within the average-risk screening pathway, whereas those with clinically relevant polyps would enter a dedicated post-polypectomy surveillance branch.

The contribution of individual predictors would change across the pathway. Before the first screening examination, clinical and EHR-derived features may provide the principal basis for risk estimation. Following colonoscopy, the number, size, histology, location, and completeness of removal of detected lesions become dominant determinants of subsequent advanced-neoplasia risk [22,23,24,25,120]. Dynamic models should therefore update predictor weighting over time rather than applying an unchanged baseline score throughout the individual’s lifetime.

At the population level, the system should monitor screening initiation and completion, adherence to repeat testing, time from a positive noninvasive result to colonoscopy, adequate bowel preparation, cecal intubation, advanced precursor detection, non-neoplastic polypectomy, surveillance assignment, interval CRC, and CRC stage at diagnosis. Model calibration and performance drift should be assessed continuously, with predefined responses when detection rates, referral completion, or subgroup performance fall below acceptable thresholds.

Equity should be embedded throughout the pathway rather than treated as a separate downstream consideration. Risk-prediction and polygenic models require validation across racial, ethnic, socioeconomic, and geographic populations before deployment [120,122,123,126]. Screening recommendations should not depend on the availability of genetic testing or advanced AI infrastructure, and lower-cost evidence-based modalities such as FIT should remain accessible default options. Blood-based tests and newer molecular assays may increase participation for some individuals, but their higher costs and lower precursor sensitivity could widen disparities if they displace established screening rather than expand access.

The deployment of CADe and digital pathology should similarly be monitored across academic, community, rural, and resource-constrained settings. AI may reduce variation by supporting less experienced clinicians and standardizing diagnostic workflows, but it may also widen existing quality gaps when access is concentrated in high-volume centers. Equity indicators should therefore include not only overall screening participation but also access to diagnostic colonoscopy, colonoscopy quality, pathology expertise, timely follow-up, and surveillance completion across patient subgroups.

Overall, the proposed pathway positions AI as a coordinating and adaptive layer across CRC screening rather than as a standalone test. EHR-based models may refine baseline risk, noninvasive biomarkers may expand participation and enrich colonoscopy yield, CADe may improve lesion recognition, and digital pathology may support reproducible classification and surveillance assignment. However, the preventive value of the pathway continues to depend on timely completion of high-quality colonoscopy and removal of clinically relevant precursor lesions. Prospective pathway-level trials are required to determine whether this integrated strategy improves advanced precursor detection, screening participation, colonoscopy efficiency, interval CRC, CRC incidence, mortality, cost-effectiveness, and equity compared with established screening programs.

## 8. Clinical Implementation and Evidence Priorities

### 8.1. Validation and Real-World Effectiveness

The translation of AI from research environments into clinical practice remains limited by heterogeneous validation and scarce prospective pathway-level evidence. Reporting frameworks such as TRIPOD+AI, STARD-AI, DECIDE-AI, and CONSORT-AI provide standards for prediction models, diagnostic accuracy studies, early clinical evaluation, and randomized trials [51,110,127,128]. Future CRC studies should align with the framework appropriate to the intended clinical role and should predefine the target population, workflow, reference standard, decision threshold, and handling of missing or uncertain data.

Methodological safeguards should also be explicit. Patient-, lesion-, slide-, and center-level data leakage must be prevented, and preprocessing, normalization, and feature selection should be performed within the training data rather than before the train–test split. Class imbalance should be addressed using class-specific metrics and precision–recall measures rather than aggregate accuracy alone. Temporal validation and geographic external validation assess different dimensions of generalizability and should not be treated as equivalent. Before external validation, the complete analytical pipeline, classifier version, and decision threshold should be locked. Subsequent updates require version control, prespecified revalidation, and post-deployment monitoring for calibration drift. For models used to determine referral to colonoscopy, AUC alone is insufficient; calibration, decision-curve or net-benefit analysis, and prospective impact evaluation are required to demonstrate improved clinical decision-making without an unacceptable false-positive colonoscopy burden [51,110,127,128,129,130]. Evaluation must extend beyond discrimination. Calibration, subgroup performance, reproducibility, external transportability, human-AI interaction, workflow effects, and clinical utility should be reported. Role-specific outcomes include advanced-neoplasia detection and false-positive colonoscopy burden for molecular screening, clinically relevant lesion yield and unnecessary polypectomy for endoscopy, and meaningful error reduction, turnaround time, and surveillance consequences for digital pathology. Predictive values and resource use should be estimated at the disease prevalence of the intended population.

Routine-practice data illustrate the difference between technical efficacy and clinical effectiveness. Additional CADe detections may be driven mainly by diminutive lesions, and performance can vary according to operator behavior, mucosal exposure, and management thresholds [97,98]. Prospective multicenter implementation studies should therefore include diverse operators, pathology laboratories, healthcare systems, and patient populations. Because molecular classifiers, endoscopic systems, CADx tools, and pathology algorithms have generally been developed independently, integrated studies must also assess cumulative error, discordant algorithmic outputs, clinician responses, and downstream management rather than evaluating each component in isolation.

### 8.2. Equity, Governance, and Regulation

AI-enabled screening raises concerns regarding data governance, privacy, algorithmic equity, clinical liability, and post-deployment oversight. Molecular classifiers, EHR models, endoscopy systems, and digital pathology platforms process sensitive data across different institutions and vendors. Governance should define data ownership, secondary use, cybersecurity, auditability, and accountability [129,130].

Algorithmic bias is a particular concern. An oncology AI review found that only 5% of studies reported the racial composition of training or validation datasets and that most participants in studies reporting race were White [126]. Models derived from demographically or geographically restricted populations may therefore be poorly calibrated in underserved settings and could widen screening disparities. External validation should include prespecified subgroup analyses and assessment of both discrimination and calibration.

Regulatory pathways must also address adaptive or periodically updated algorithms. Post-deployment monitoring, version control, performance drift, and the degree of revalidation required after modification should be explicit. Responsibility for erroneous or missed AI-assisted decisions remains distributed across developers, institutions, and clinicians, reinforcing the need for human oversight and transparent documentation [129,130].

### 8.3. Cost-Effectiveness and Colonoscopy Burden

The economic value of AI-enhanced screening depends on the complete pathway rather than the acquisition price of a test or software platform. Modeling suggests that blood-based screening may be preferable to no screening but is often less cost-effective than established stool-based or colonoscopic strategies when adherence is assumed to be comparable [131]. Its value may therefore depend on reaching individuals who would otherwise remain unscreened.

Economic evaluations of CADe must include licensing and equipment costs, additional polypectomy and pathology, complications, and changes in surveillance intervals. Digital pathology may reduce workload and improve reproducibility, but savings must be balanced against scanners, storage, integration, validation, and maintenance. Cost-effectiveness analyses should use realistic completion rates for every step, particularly diagnostic colonoscopy after a positive noninvasive test.

### 8.4. Future Research Priorities

The highest research priority is prospective comparison of complete screening pathways rather than further isolated cancer-versus-control classifier development. Studies should determine whether integrated risk assessment and multimodal testing improve participation and advanced precursor detection without creating unacceptable colonoscopy demand, unnecessary intervention, or surveillance burden. Head-to-head comparisons should use common definitions for advanced adenomas and SSLs and should report lesion-specific performance, referral completion, complications, interval CRC, CRC stage, cost-effectiveness, and equity.

Federated learning may support collaboration across institutions without centralizing raw patient data, but it does not automatically eliminate bias, heterogeneity, or privacy risk. Harmonized variables, common outcomes, secure governance, and independent external validation remain necessary. Ultimately, randomized or pragmatic trials must determine whether multimodal AI-supported screening reduces interval CRC, CRC incidence, and mortality compared with established screening programs. Table 5 translates these evidence gaps into modality-specific evaluation requirements and patient-relevant outcomes for future research.

## 9. Conclusions

The precancerous gap provides a clinically useful framework for evaluating innovation in CRC screening. Current blood-based assays can increase access and detect established CRC, but their low sensitivity for advanced adenomas and SSLs limits their ability to reproduce the preventive effect of colonoscopy. Fragmentomic, multi-omic, and RNA-based approaches may improve precursor detection, although the most favorable results frequently derive from enriched or case–control cohorts and require independent prospective validation.

AI-assisted endoscopy has the strongest clinical evidence and consistently improves ADR and adenoma miss rates. Its effect on advanced neoplasia, serrated lesions, interval CRC, incidence, and mortality remains uncertain, and additional detection may increase low-value polypectomy and surveillance. Digital pathology can improve classification and prioritize high-risk specimens but acts only after a lesion has been detected and sampled.

The most plausible clinical future is therefore not replacement of established screening modalities but coordinated, risk-adapted use. EHR models may refine baseline risk, noninvasive tests may improve participation and molecular risk estimation, CADe may support lesion recognition, and digital pathology may standardize post-resection classification. High-quality colonoscopy and complete polypectomy should remain the central preventive intervention until integrated pathways demonstrate superior patient-derived outcomes in diverse prospective populations.

## Figures and Tables

**Table 1:** Current and emerging CRC screening modalities.

Modality	Main Clinical Strength	Advanced Precursor Performance	Principal Limitation	References
Colonoscopy	Direct visualization and immediate polypectomy	Highest sensitivity, approximately 89–95% for adenomas at least 10 mm	Participation, access, bowel preparation, operator-dependent miss rate	[9,10,33]
FIT	Low cost, scalable, home-based testing	Approximately 23–28% sensitivity for advanced adenomas	Bleeding-dependent signal and repeated testing required	[10,35]
Multitarget stool DNA	Combines occult blood and molecular markers	Approximately 43–45% sensitivity for advanced precancerous lesions	Lower specificity and greater colonoscopy burden than FIT	[34,35]
Multitarget stool RNA	Combines RNA markers with FIT	Approximately 46% sensitivity for advanced adenomas in a prospective screening cohort	Longitudinal effectiveness and optimal interval require further study	[36]
Blood-based cfDNA	High acceptability and clinically relevant CRC sensitivity	Approximately 13% sensitivity for advanced precancerous lesions in prospective average-risk cohorts	Limited preventive capacity and dependence on follow-up colonoscopy	[11,12,37,38]

Abbreviations: cfDNA, cell-free DNA; CRC, colorectal cancer; FIT, fecal immunochemical test.

**Table 2:** Representative noninvasive molecular approaches for CRC and advanced precursor detection.

Approach	Biological Signal	Representative Study and Sample Size	Study Design and Validation Type	Representative Performance	Main Limitation	References
Single-gene cfDNA methylation	Methylated *SEPT9*	PRESEPT: *n* = 7941 enrolled; *n* = 1516 selected for assay analysis *n* = 1510 valid	Prospective, multicenter screening validation in asymptomatic average-risk adults; stratified random assay subsample; colonoscopy reference standard	48.2% CRC and 11.2% advanced adenoma sensitivity; 91.5% specificity	Low precursor and early-stage sensitivity; performance estimated from an assay subsample	[54]
Multimodal cfDNA assay	Genomic alterations, aberrant methylation, and fragmentomic patterns	ECLIPSE: *n* = 10,258 in the clinical-validation cohort; *n* = 7861 evaluable	Prospective, multicenter clinical validation in an average-risk screening population; colonoscopy reference standard	83.1% CRC and 13.2% advanced precancerous lesion sensitivity; 89.6% specificity for advanced neoplasia	Strong CRC detection but weak precursor sensitivity; single-round accuracy without incidence or mortality outcomes	[11]
Targeted cfDNA methylation assay	CRC-associated CpG methylation patterns	PREEMPT CRC: *n* = 48,995 enrolled; *n* = 27,010 evaluable	Prospective, multicenter, cross-sectional validation in an average-risk screening population; blinded testing with colonoscopy and histopathology as the reference standard	79.2% CRC and 12.5% advanced precancerous lesion sensitivity; 91.5% specificity for advanced neoplasia	Low advanced precursor sensitivity, particularly for sessile serrated lesions; longitudinal effectiveness remains unknown	[37]
cfDNA fragmentomics	Genome-wide fragment lengths, end motifs, and coverage	Hong et al.: *n* = 1677; development cohort *n* = 1250 validation cohort *n* = 427	Prospective case–control study with a random within-study development–validation split; no independent external validation	Validation cohort: 90.4% CRC and 58.3% advanced adenoma sensitivity; 94.7% specificity	Case–control spectrum and within-study validation limit generalizability to average-risk screening	[61]
Multimodal cfDNA-based assay	Genomic, epigenomic, and fragmentomic cfDNA features; plasma proteins in the refined version	Bessa et al.: *n* = 623	Disease-enriched two-cohort evaluation involving FIT-positive screening participants and patients with established CRC; no independent external validation	93% CRC sensitivity and 90% specificity; 23% advanced precursor sensitivity with the refined version	Spectrum bias, assay and threshold refinement, and limited precursor detection	[65]
Blood transcriptomic–protein model	29-gene expression panel in peripheral blood mononuclear cells combined with plasma tumor markers	Ciarloni et al.: development *n* = 181; independent validation *n* = 168; additional cases with other colorectal findings or non-CRC diseases *n* = 245	Multicenter case–control development followed by independent hold-out testing; not validated prospectively in an average-risk screening population	78.1% CRC and 52.3% large adenomatous polyp sensitivity; 92.2% specificity	Potential host-response and comorbidity confounding; case–control spectrum and absence of population-level screening validation	[67,68]
Multitarget stool RNA–FIT classifier	Eight stool RNA transcripts, FIT, and participant-reported smoking status	CRC-PREVENT: *n* = 8920 eligible participants	Phase III, blinded, prospective, cross-sectional validation in an average-risk screening population; colonoscopy reference standard	94.4% CRC and 45.9% advanced adenoma sensitivity; 87.9% specificity for no lesions on colonoscopy	Lower specificity than FIT; optimal repeat interval and long-term effectiveness remain undetermined	[36]

Reported estimates are not directly comparable because populations, reference standards, and lesion definitions differ.

**Table 4:** Representative applications and intended clinical roles of AI in colorectal digital pathology.

Intended Clinical Role	Representative Evidence	Near-Term Clinical Value	Main Limitation	References
Standalone or autonomous multiclass classification (investigational)	External classification of common polyp subtypes across 24 institutions; additional multiclass dysplasia and carcinoma models	Standardization of diagnostic classification	Errors in overlapping categories and incomplete external validation of the full multiclass task	[16,105,111]
AI-assisted decision support or second reading	Interactive classification increased pathologist accuracy from 73.9% to 80.8%; dedicated SSL-support systems achieved expert-comparable performance	Improved consistency and identification of uncertain or potentially discordant slides while retaining pathologist oversight	Interface dependence, automation bias, and over-reliance on algorithmic output	[102,103,104]
Binary triage and case prioritization	In a large external evaluation, 98.9% of polyps with high-grade dysplasia or adenocarcinoma were assigned to specialist review	Faster review of high-risk slides and potential workload reduction	False-negative risk, alert fatigue, threshold dependence, and domain shift	[107,108,109]
Tumor detection and tissue segmentation	High slide-level tumor sensitivity across multiple pathology departments	Highlighting suspicious regions and supporting targeted review	Variation in staining, scanner platforms, tissue preparation, and local diagnostic criteria	[106,111]

**Table 5:** Clinical implementation challenges and corresponding evidence priorities.

Domain	Current Problem	Minimum Evaluation Requirement	Patient-Relevant Outcome
Molecular biomarkers	Spectrum bias and low precursor shedding	Prospective average-risk cohorts with lesion-specific reporting	Advanced adenoma and SSL detection; colonoscopy burden
EHR risk models	Calibration drift and incomplete transportability	External validation across populations and health systems	Screening completion and advanced-neoplasia yield
CADe and CADx	Surrogate endpoint emphasis and over-detection	Pragmatic multicenter studies with workflow and harm measures	Interval CRC, unnecessary polypectomy, incidence, and mortality
Digital pathology	Scanner, stain, and laboratory domain shift	Prospective human–AI studies across laboratories	Clinically important error rate and turnaround time
Integrated pathways	Independent development of individual components	Pathway-level trials assessing cumulative error and discordance	Participation, prevention, equity, and cost-effectiveness

## Data Availability

No new data were generated or analyzed in this study. Data sharing is not applicable to this article.
